# Exploring the Impact of Primer–Template Mismatches on PCR Performance of DNA Polymerases Varying in Proofreading Activity

**DOI:** 10.3390/genes15020215

**Published:** 2024-02-07

**Authors:** Ke Huang, Jilei Zhang, Jing Li, Haixiang Qiu, Lanjing Wei, Yi Yang, Chengming Wang

**Affiliations:** 1 College of Veterinary Medicine, Yangzhou University, Yangzhou 225012, China; huangke126@126.com (K.H.); 20221030@wnmc.edu.cn (J.L.); qiulihx@126.com (H.Q.); 2 Center of Animal Disease Control and Prevention, Songjiang District, Shanghai 201600, China; 3 College of Medicine, University of Illinois Chicago, Chicago, IL 60607, USA; jileiz@uic.edu; 4 Bioengineering Program, The University of Kansas, Lawrence, KS 66045, USA; lanjingwei@ku.edu; 5 Department of Pathobiology, College of Veterinary Medicine, Auburn University, Auburn, AL 36849, USA

**Keywords:** PCR, DNA Polymerases, nucleotide mismatch, Proofreading

## Abstract

Polymerase chain reaction (PCR) is a widely used technique in gene expression analysis, diagnostics, and various molecular biology applications. However, the accuracy and sensitivity of PCR can be compromised by primer–template mismatches, potentially leading to erroneous results. In this study, we strategically designed 111 primer–template combinations with varying numbers, types, and locations of mismatches to meticulously assess their impact on qPCR performance while two distinctly different types of DNA polymerases were used. Notably, when a single-nucleotide mismatch occurred at the 3’ end of the primer, we observed significant decreases in the analytical sensitivity (0–4%) with Invitrogen™ Platinum™ *Taq* DNA Polymerase High Fidelity, while the analytical sensitivity remained unchanged with Takara Ex *Taq* Hot Start Version DNA Polymerase. Leveraging these findings, we designed a highly specific PCR to amplify *Babesia* while effectively avoiding the genetically close *Theileria*. Through elucidating the critical interplay between types of DNA polymerases and primer–template mismatches, this research provides valuable insights for improving PCR accuracy and performance. These findings have important implications for researchers aiming to achieve robust qPCR results in various molecular biology applications.

## 1. Introduction

The invention of the polymerase chain reaction (PCR) by Kary B. Mullis in the 1980s [1,2,3,4] marked a seminal moment in molecular biology, comparable in impact to the revelation of the double-helix structure of DNA and DNA cloning. The multitude of variations derived from this elegant approach to sequence-specific and in vitro exponential DNA replication has opened new avenues of scientific inquiry in biology, revolutionizing molecular biology and medical diagnosis.

The sensitivity and specificity of PCR in nucleic acid detection are influenced by various factors, including primer design, PCR buffer/additives, single-nucleotide polymorphisms (SNPs) and nucleotide differences in the target genes, and the properties of DNA polymerases and the thermal protocols [5,6]. Ideally, designed primers should perfectly complement the target template. However, this can be challenging as mutations in the target genes are often present. Particularly in cases where PCR is designed to amplify multiple similar targets or in duplex and multiplex PCR systems in a single reaction system, achieving 100% nucleotide complementarity between primers and target templates is practically unattainable. Therefore, gaining a comprehensive understanding of the effects of different types of primer–template mismatches is crucial to designing appropriate primers that ensure the sensitivity, specificity, and amplification efficiency of PCR.

DNA polymerase plays an essential role in PCR by synthesizing new DNA strands. Fidelity, specificity, thermostability, and processivity are critical characteristics in evaluating DNA polymerase [7,8,9]. Fidelity refers to the accuracy of DNA sequence replication and is determined by the proofreading capability of a DNA polymerase. High-fidelity DNA polymerases possess strong proofreading activity [8].

While several studies have investigated the effects of primer mismatches, mainly at the 3’ end of the primer, on PCR performance [9,10,11,12,13,14,15,16,17], few have systematically examined the effects of different types, numbers, and locations of nucleotide mismatches on the amplification efficiency of PCR. In this study, we address this gap by investigating the collaborative effects of the different types of DNA polymerases and nucleotide mismatches on PCR performance. Leveraging a highly sensitive quantitative PCR platform, we comprehensively evaluated the impact of 111 different types of nucleotide mismatches in the primers, utilizing two distinctly different types of DNA polymerases.

## 2. Materials and Methods

### 2.1. Establishment of a Chlamydia pneumonia FRET-PCR System

The 168 bp fragment of the real-time fluorescence resonance energy transfer (FRET) qPCR targeting the *Chlamydia pneumoniae* 23S rRNA gene followed the previous design with minor modifications [18,19]: upstream primer: 5’-GGGGTTGTAGGGTCGATAACGTGAGATC-3’; downstream primer: 5’-GAGAGTGGTCTCCCCAGATTCAGACTA-3’; 6-FAM probe: 5’-GAACGATACAGGGTGATAGTCCCGTA-6-FAM-3’; LCRed640 probe: 5’-LCRed640-ACGAAAAAACAAAAGACGCTAATCGAT-phosphate-3’.

The FRET-qPCR for *Chlamydia* spp. was conducted on a LightCycler 480-II real-time PCR platform, and thermal cycling consisted of 18 high-stringency step-down cycles followed by 30 relaxed-stringency fluorescence acquisition cycles. The 18 high-stringency step-down thermal cycles were 6 × 15 s @ 95 °C, 60 s @ 72 °C; 9 × 15 s @ 95 °C, 60 s @ 70 °C; 3 × 15 s @ 95 °C, 30 s @ 68°C, 30 s @ 72 °C. The relaxed-stringency fluorescence acquisition cycling consisted of 30 × 15 s @ 95 °C, 8 s @ 56 °C followed by fluorescence acquisition, 30 s @ 65 °C, and 30 s @ 72 °C. The PCR master mix contained upstream primer (1.0 μM), downstream primer (1.0 μM), and two probes (0.2 μM), and the PCR reaction buffer and other conditions were as described [18].

The PCR reaction buffer was 4.5 mM MgCl_2_, 50 mM KCl, 20 mM Tris (hydroxymethyl aminomethane)-HCl, pH 8.4, supplemented with 0.05% each Tween 20 and Nonidet P-40, and 0.03% acetylated BSA (Roche Applied Science). Nucleotides were used at 0.2 mM (dATP, dCTP, dGTP) and 0.6 mM (dUTP). For each 20 μL total reaction volume, we used 1.5 U Taq DNA polymerase [18].

The plasmid manufactured with the pUC57 cloning vector (GenScript, Nanjing, China) containing the 168 bp fragment of the 23S rRNA gene of *C. pneumoniae* were used as quantitative standards. The molarity of the amplicon-contained plasmid was determined and adjusted to provide solutions containing 10,000, 1000, 100, 10, and 1 gene copies per PCR reaction in 1 × T_10_E_0.1_ buffer as quantitative standards in this study. These were amplified in duplicate to determine the detection limit of the PCR. The copy numbers were calculated based on dilutions of quantitative standards, which were determined based on their molarity. The negative control with DNA/RNA-free H_2_O was included in all the PCR reactions.

The established FRET-qPCR was tested on the standards (10,000, 1000, 100, 10, and 1 gene copies per PCR reaction) when seven different DNA polymerases were used to test the specificity and reproducibility: Platinum^®^*Taq*DNA polymerase, Invitrogen, Carlsbad, CA, USA, 10966-018, 8215130; TaKaRa Ex *Taq*^®^Hot Start Version, Takara, Japan, RR06WZ, RR006Q; FastStart *Taq* DNA Polymerase, Roche, USA, Indianapolis, IN 12032902001, 13860081; Phanta^TM^ HS Super-Fidelity DNA Polymerase, Vazyme, Germany, P502, 013041; Phanta^TM^ Super-Fidelity DNA Polymerase, Vazyme, Germany, P501, 017031; Ace*Taq*^TM^ HS DNA Polymerase, Vazyme, P401, 425031; Chimerigen Laboratories *Taq* DNA Polymerase, Chimerigen, Germany, TG102, D1208001 (Figure S2). This study complied with the MIQE guidelines guaranteeing minimum information for publication of quantitative real-time PCR experiments [20] (Table S1).

### 2.2. Nucleotide Mismatches in the Primers

To investigate the impact of nucleotide mismatches on PCR amplification efficiency, we conducted several experiments. First, we introduced 34 types of single-nucleotide mismatches at the 3’ end of the primer (Table 1). Additionally, we generated 40 types of 2–5 nucleotide mismatches at the 3’ end of the primer (Table 2). Furthermore, we created 9 types of mismatches in the center and 5’ end of the primers (Table 3). Lastly, we generated 28 types of mismatches involving both the upstream and downstream primers (Table S2).

### 2.3. Calculation of the Relative PCR Amplification Efficiency

The *C. pneumoniae* quantitative standards, consisting of 10,000, 1000, 100, 10, and 1 gene copies per PCR reaction, were initially tested in the PCR system without any nucleotide mismatches. The obtained equation from this mismatch-free PCR system was then utilized to determine the copy numbers of the quantitative standards in the PCR systems with different types of nucleotide mismatches. To calculate the relative amplification efficiency for a specific PCR system with a mismatch, the following formula was employed:

(Relative copy number for the 100-copy standard/100 + Relative copy number for the 10-copy standard/10) × 50%.

By applying this formula, the relative amplification efficiency of the PCR system with the nucleotide mismatch could be determined.

### 2.4. Establishment of Babesia PCR to Differentiate Babesia and Theileria

To design primers specific for *Babesia*, the 18S rRNA sequences of *Babesia* and *Theileria* were obtained from GenBank. The *Babesia* sequences include *B. bigemina* (GenBank Accession number JQ723014), *B. motasi* (AY260179), *B. crassa* (AY260176), *B. canis* (AY072926), *B. vogeli* (HM590440), *B. rossi* (DQ111760), *B. capreoli* (FJ944828), *B. divergens* (FJ944822), *B. odocoilei* (AY661508), and *B. hongkongensis* (JQ867356). *Theileia* species include *T. caprech* (AY726011), *T. ovis* (AY260172), *T. buffeli* (AY661512), *T. annulata* (FJ26369), *T. annulata* (M64243), and *T. equi* (Z15105). In addition, the 18S rRNA sequences genetically close to *Babesia* were also collected: *Toxoplasma gondii* (L37415), *Neosporo caninum* (U63069), *Eimeria arnyi* (AY613853), *Cytauxzoon felis* (AY679105), *Hepatozoon americanum* (AF176836), *Cryptosporidium meleagridis* (AF112574), and *C. parvum* (L16996).

These sequences were aligned to design primers specific for *Babesia*, introducing different types of nucleotide mismatches with those of *Theileria*. The plasmid manufactured with the pUC57 cloning vector (GenScript, Nanjing, Jiangsu, China) containing fragments of PCR amplicons was used to generate quantitative standards (with copy numbers of 10^3^, 10^2^, 10^1^, and 10^0^) for both *Babesia* and *Theileria*.

In the literature, two commonly used published PCR primer sets for *Babesia* were identified, the first by Olmeda et al. [20] (1023 citations based on Google Scholar, accessed on 30 July 2023) and the second by Casati et al. [21] (328 citations based on Google Scholar, accessed on 30 July 2023). Along with the primers designed in this study, these primer sets from Olmeda et al. and Casati et al. [20,21] were used to test the presence of *Babesia* DNA in the blood of goats in China (n = 40). The PCR products were analyzed with gel electrophoresis, followed by DNA sequencing with the upstream and downstream primers in the same PCR (GenScript, Nanjing, China). The experiments were performed in duplicate, comparing the detection of *Babesia* and *Theileria* using the published primers and the system established in this work.

## 3. Results

### 3.1. Establishment of a Highly Sensitive Quantitative PCR

The quantitative standards, consisting of 10^4^, 10^3^, 10^2^, 10^1^, and 10^0^ copies of the gene per PCR reaction, were tested using the established quantitative PCR system with the use of two different DNA polymerases. When Platinum and Takara DNA polymerases were employed, the PCR system reliably amplified the single copy of the gene target (Figure S1) in duplicate. For the other five types of DNA polymerases, the PCR systems exhibited detection limits ranging from 10 to 1000 copies (Figure S2). In this study, Platinum and Takara DNA polymerases were utilized to evaluate the sensitivity and specificity of the PCR amplification in the presence of nucleotide mismatches in the primers.

### 3.2. A Single-Nucleotide Mismatch at 3′ Primer Reduced the Amplification Efficiency When Platinum, but Not Takara, Was Used

**Figure 1:**
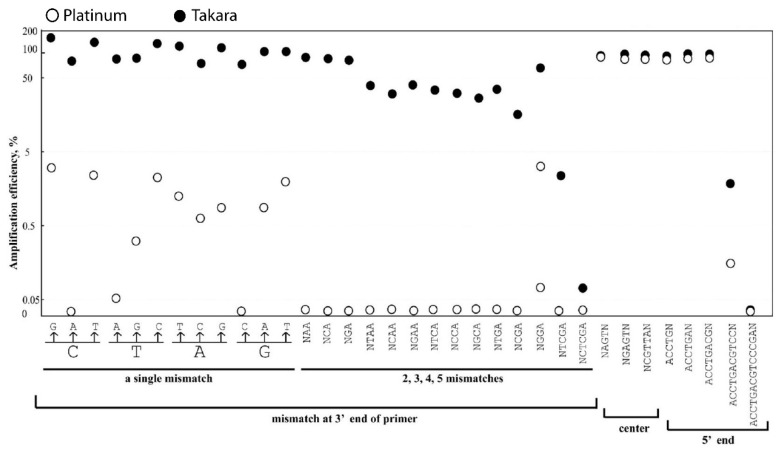
**Amplification efficiencies of *C. pneumoniae* PCR affected by nucleotide mismatches and different DNA polymerases**. The abscissa shows the different positions, numbers, and types of nucleotide mismatches, and the ordinate indicates the PCR amplification efficiencies. Two types of DNA polymerases were tested and compared. N indicates the nucleotide of A, T, C, or G.

**Figure 2:**
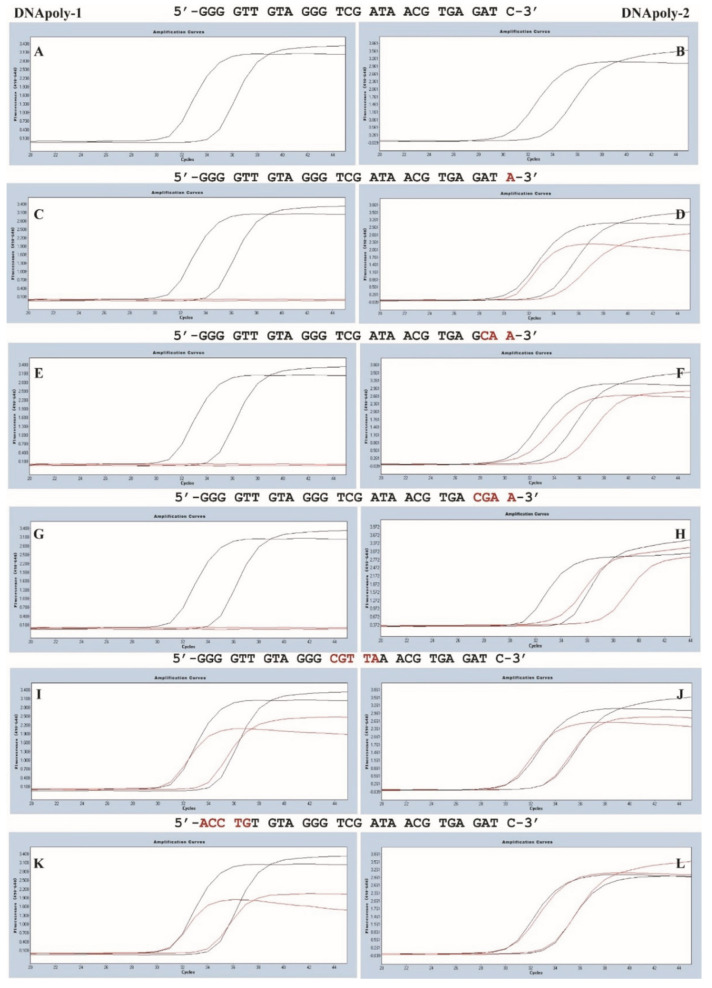
**Amplification curves of qPCRs with nucleotide mismatches in the primers using two types of DNA polymerases.** PCR amplification curves free of nucleotide mismatches in the primers with two types of DNA polymerases (**A**,**B**). Amplification curves of PCRs with nucleotide mismatches in 3′ end (**C**–**H**), center of the primers (**I**,**J**), and 5′ end of the primers (**K**,**L**). The abscissa represents cycles and ordinate reveals the fluorescence intensity. The curve demonstrates the 100 and 10 copies of *Chlamydia* 23S rRNA gene. Black curves: perfect match; red curves: mismatch. The y-axis denotes the log scale of the ratios for the fluorescent signal in two channels (F4/F1). For both black and red curves, the left one with lower ct value indicates 100 copies and the other one denotes 10 copies.

To observe the impact of a single-nucleotide mismatch on amplification efficiency, a comprehensive set of 12 nucleotide mismatches was generated in the 3’ end of the upstream primer, including C to G, A, and T; T to A, G, and C; and A to T, G, and C (Table 1). When Platinum was used, a single-nucleotide mismatch in the upstream primer caused a drastic decrease in amplification efficiency, dropping from 100% to 0–4% (Table 1; Figure 1 and Figure 2). In contrast, when Takara was used, the amplification efficiency remained the same or even increased unexpectedly with a single 3’ primer mismatch (Table 1; Figure 1 and Figure 2).

Similarly, when degeneracy mismatches were introduced at the 3’ end of the primer, the amplification efficiency remained at 100% for Takara, but dropped to 34–63% with Platinum (Table 1).

When a single-nucleotide mismatch occurred in the second to fourth position of the 3’ end of the primer, a significant drop in amplification efficiency, even down to 0%, was observed with Platinum. However, the amplification efficiency for Takara remained above 85% (Table 1; Figure 1).

When two, three, or five mismatches were generated in the 3’ end of the primers, the amplification efficiency dropped to 0% for Platinum, except for a 10% efficiency observed for the ATC to GAT mismatch (Table 2; Figure 1; Table S2). In comparison, for Takara, two mismatches had only a marginal influence on the amplification efficiency, while four and five mismatches reduced the efficiency to 0–14% (Table 2; Figure 1; Table S2).

### 3.3. Nucleotide Mismatch in the Center and 5’ End of the Primer Influences the Amplification Moderately

The introduction of three to five mismatches in the center of the primer, as well as up to eight mismatches in the 5’ end of the primer, resulted in a slight reduction in amplification efficiency (up to 82%) for both Platinum and Takara DNA polymerases. However, when more than 11 nucleotide mismatches were present, the PCR amplification was completely prevented (Figure 1; Table S2).

### 3.4. Amplification Efficiencies Affected by Nucleotide Mismatches in Upstream Primer, Center, and Downstream of the Primer

When mismatches were present at both ends of the upstream and downstream primers, the amplification efficiency dropped to 0% for Platinum and decreased to up to 19% for Takara (Table S3; Figure S3). However, when multiple mismatches occurred in the center and 5’ end of the primer, the amplification efficiency remained above 44%.

Interestingly, when a 3’ downstream mismatch (A to T) was combined with multiple mismatches in the 5’ and center of the upstream primer, the amplification efficiency remained at 74% and 67% (Table S3; Figure S3). This finding highlights the complex interplay between different mismatch positions and their effects on amplification efficiency.

### 3.5. Designed Babesia PCR Specifically Amplifies Babesia, but Not Theileria

**Figure 3:**
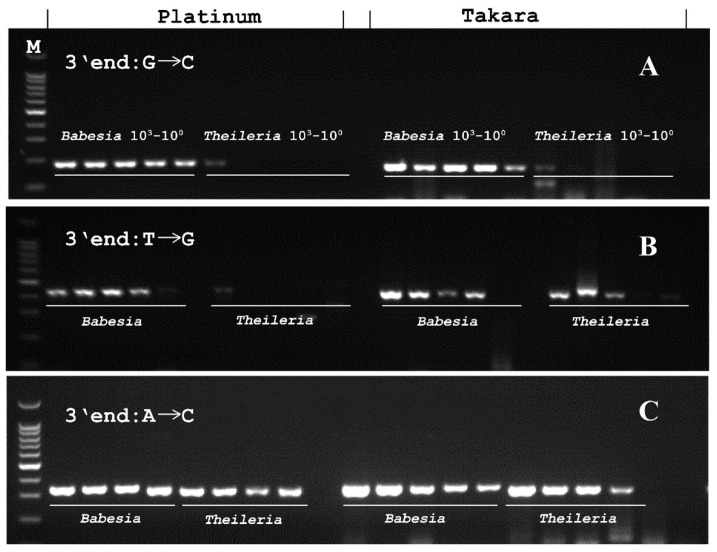
***Babesia* PCR with different primers and DNA polymerases to amplify *Babesia* and *Theileria*.** (**A**) (PCR-5), (**B**) (PCR-1), and (**C**) (PCR-4, 2, 3) indicated three different PCR primers which have different single-nucleotide mismatch at the 3’ end between *Babesia* and *Theileria* 18S rRNA sequences. Platinum DNA and Takara DNA polymerases were used. The samples were 1000, 100, 10, and 1 gene copies for both pathogens, while the samples of 1000, 100, 10, and 1 gene copies were used in row C (Platinum).

In this study, five sets of primers were designed and tested to amplify 10^3^, 10^2^, 10^1^, and 10^0^ copies of *Babesia* and *Theileria* 18S rRNA (Table 3; Figure 3).

Among these primers, PCR-5 had a 3’ end nucleotide mismatch from C to G between the *Babesia* and *Theileria* 18S sequences (Table 3). In this PCR system using Platinum, a weak amplification was observed with 1000 copies, but not 100, 10, and 1 copies of *Theileria*. Conversely, with Takara used, a weak amplification of 1000 and 100 copies of *Theileria* DNA was observed (Figure 3).

In PCR-1 which had a 3’ end mismatch from G to T, specific amplification was observed only for *Babesia* targeted with Platinum. However, the strong amplification of 1000, 100, and 10 copies of *Theileria* occurred when Takara was used (Figure 3).

Similar amplification patterns were observed in PCRs 2, 3, and 4, which had other types of nucleotide mismatches (Table 3; Figure 3). Strong amplifications of up to a single copy of *Theileria* DNA were observed using both enzymes (Figure 3).

The PCR-5, along with two commonly used published primers (Table 3), was used to test 40 blood samples from goats with potential tick-borne agents including *Babesia*, *Theileria,* and *Ehrlichia*. The primer system from Olmeda et al. [21] identified twelve samples as *Babesia* positive, but DNA sequencing confirmed that only one out of these twelve samples was truly *Babesia*, while eight were *Theileria*, and three had hybrid sequences. The primer system from Casati et al. [22] identified twenty samples as *Babesia* positive, but DNA sequencing confirmed that only one out of these twenty samples was *Babesia*, while seventeen were *Theileria*. In comparison, the PCR-5 established in this study identified three samples as positive for *Babesia*, and DNA sequencing confirmed that all three of these samples were indeed *Babesia* positive.

## 4. Discussion

While many studies have reported that primer mismatches can lead to a reduction in PCR amplification efficiency [9,10,11,12,13,14,15,16], the extent of this reduction has shown significant variability across studies. Some studies even suggested that a single mismatch at the primer’s 3′ end had little to no impact on qPCR yield [14,17]. The discrepancies in reduced efficiencies may stem from various factors, such as differences in thermal protocols, amplicon size, amplification cycles, master mixes, and DNA polymerases used. To address this question quantitatively, we employed a highly sensitive qPCR system while keeping the entire reaction system and reagents consistent, thereby focusing solely on the impact of primer mismatches and different DNA polymerases.

Regarding the primer mismatches, we designed 111 different types of mismatches with varying numbers [1,2,3,4,5] and locations on one primer (3′ end, near the 3′ end, center of the primer, 5′ end) or both primers, as well as diverse mismatch types (full combinations of A, T, C, and G mismatches and degeneracy). We observed a significant decrease in amplification efficiency when single mismatches were introduced at the first (0–4%), second (0–13%), and third (5–12%) bases from the 3’ terminal of the primer using Platinum. However, this mismatch effect diminished when mismatches were located >4 base pairs away from the 3’ end of the primer (Table 1; Figure 1). Moreover, complete amplification blockage occurred when >1 continuous mismatches were introduced at the 3’ end of the primer (Table 2). Interestingly, even a single degenerate at the 3’ end of the primer still allowed for acceptable amplification efficiency (34–63%) (Table 1). Conversely, the performance of Takara differed. Single mismatches or degenerates, as well as double mismatches at the 3’ end of the primer, resulted in amplification efficiencies of almost or even above 100% (Table 1 and Table 2), except when >2 continuous mismatches were present (Table 2).

The availability of various DNA polymerases with different fidelity levels on the market for PCR introduces additional complexity. Pol I DNA polymerases retain 5’–3’ exonuclease activity but not 3’–5’ exonuclease activity, such as with the Taq DNA polymerase, while α-type DNA polymerases retain 3’–5’ exonuclease activity but not 5’–3’ exonuclease activity, such as in the cases of the KOD and Pfu DNA polymerases. Both enzymes used in this study belong to Pol I DNA polymerase, but the presence or absence of proofreading activities within these enzymes provides a clear explanation for their markedly distinct responses to primer–template mismatches observed in this research.

One limitation of this study was that the fidelity levels of two types of Taq DNA polymerases used in this study were not evaluated in comparison to the Pol I DNA polymerases. Variants of a *Thermus aquaticus* DNA Polymerase were found to show an increased selectivity for applications in allele- and methylation-specific amplification [23]. Similarly, Lim et al. (2022) reported a modified *Taq* DNA polymerase for allele-specific ultra-sensitive detection of genetic variants [24].

Two DNA polymerases tested in this study were found to amplify one copy of *C. pneumoniae* 23S rRNA and were also further analyzed to ascertain their impact on PCR performance along with different types of primer–template mismatches. Our study emphasizes the importance of avoiding 3’ primer mismatches, especially G to C, C to A, T to G, and T to A, in PCR design to prevent false-negative results when using Platinum. Furthermore, the results from this work could also be used to improve the selectivity of PCR assays so that primers are designed to have a perfect match on the intended target, particularly the 3′ primer mismatches on unintended sequences, to avoid off-target amplification of homologous or orthologous sequences. However, it is noteworthy that we observed amplification efficiencies over 100% with the 3’ primer mismatches G to C and T to C when Takara was used.

This study revealed that certain mismatches can still lead to amplification, as evidenced by the higher amplification efficiency observed in the case of ATC compared to GAT. This phenomenon could be attributed to several factors. First, it may be a consequence of the upstream primer containing a GAT sequence, which introduces a wobble effect at the beginning. Second, the efficiency of mismatches involving C > T and ATC > TCT may stem from the fact that the first base following the primer is a T. In situations where a terminal mismatch is present in the primer, it results in a bulge formation at the penultimate position.

Drawing on our findings, we designed five sets of PCRs to amplify *Babesia*, evaluating their application efficiency alongside two commonly used, published *Babesia* primers [21,22]. The results obtained from quantitative standards and clinical samples validated the findings of our study.

In this study, we determined the relative amplification efficiency of the PCR by calculating the ratio of copy numbers in the tested system with and without nucleotide mismatches. However, for a more precise assessment of PCR amplification efficiency in future studies, it is highly recommended to use the expression E = 10^(−1/s)^, where s is the slope of the trend line which fits the curve plotting Ct values against the corresponding standard concentrations expressed in a logarithmic scale [25]. Alternatively, efficiency can be analyzed by using the raw data of qPCR with LinReg software [26,27]. Analytical microbiology, as exemplified by ISO/TS 12869 for water quality, permits a broad spectrum of PCR efficiency variation (75–125%). Consequently, the choice of method for calculating PCR efficiencies can significantly influence quantitative results and detection limits. Given this, adopting a method that involves utilizing the slope of each individual PCR reaction to calculate PCR efficiency can promote universality across different laboratories. This standardized approach aims to minimize variations between different labs.

Another limitation of this study was the reliance on the mean of the PCR efficiency in duplicate. Ideally, statistical comparisons would be strengthened by analyzing the mean along with standard deviations, leading to more robust and reliable conclusions.

In conclusion, this study highlights the critical impact of both primer–template mismatches (including their types, numbers, and locations) and the properties of DNA polymerase on the quantitative measurement of DNA by PCR. To ensure highly sensitive and specific amplification, it is crucial to use DNA polymerase supplemented with proofreading activities while avoiding 3’ primer mismatches such as G to C, C to A, T to G, and T to A. Given the comprehensive assessment of 111 primer–template mismatches using two different DNA polymerases in this study, our conclusions are likely to contribute to the improvement of qPCR design and the accuracy of molecular diagnostics. Researchers in various molecular biology applications can benefit from these findings to achieve more reliable and robust qPCR results.

## Figures and Tables

**Table 1:** Amplification efficiency influenced by a single-nucleotide mismatch at 3’ end of the primers.

Nucleotide Mismatch	Amplification Efficiency	
GGGGTTGTAGGGTCGATAACGTGAGATC	Platinum, 100%	Takara, 100%
GGGGTTGTAGGGTCGATAACGTGAGATG *	4%	190%
GGGGTTGTAGGGTCGATAACGTGAGAT**A**	0%	90%
GGGGTTGTAGGGTCGATAACGTGAGAT**T**	3%	165%
GGGGTTGTAGGGTCGATAACGTGAGA**A**	0%	100%
GGGGTTGTAGGGTCGATAACGTGAGA**G**	0%	100%
GGGGTTGTAGGGTCGATAACGTGAGA**C**	3%	160%
GGGGTTGTAGGGTCGATAACGTGAG**T**	1%	150%
GGGGTTGTAGGGTCGATAACGTGAG**C**	2%	95%
GGGGTTGTAGGGTCGATAACGTGAG**G**	1%	130%
GGGGTTGTAGGGTCGATAACGTGA**C**	0%	80%
GGGGTTGTAGGGTCGATAACGTGA**A**	1%	115%
GGGGTTGTAGGGTCGATAACGTGA**T**	2%	120%
GGGGTTGTAGGGTCGATAACGTGAGATM **	59%	100%
GGGGTTGTAGGGTCGATAACGTGAGAT**S**	56%	100%
GGGGTTGTAGGGTCGATAACGTGAGAT**Y**	63%	100%
GGGGTTGTAGGGTCGATAACGTGAGAT**H**	48%	100%
GGGGTTGTAGGGTCGATAACGTGAGAT**V**	39%	100%
GGGGTTGTAGGGTCGATAACGTGAGAT**B**	43%	100%
GGGGTTGTAGGGTCGATAACGTGAGAT**N**	34%	100%
GGGGTTGTAGGGTCGATAACGTGAGA**G**C	0%	90%
GGGGTTGTAGGGTCGATAACGTGAGA**A**C	1%	93%
GGGGTTGTAGGGTCGATAACGTGAGA**C**C	13%	95%
GGGGTTGTAGGGTCGATAACGTGAG**C**TC	12%	92%
GGGGTTGTAGGGTCGATAACGTGAG**T**TC	12%	92%
GGGGTTGTAGGGTCGATAACGTGAG**G**TC	5%	85%
GGGGTTGTAGGGTCGATAACGTGA**T**ATC	37%	98%
GGGGTTGTAGGGTCGATAACGTG**C**GATC	82%	100%

* Text with bold indicates a nucleotide mismatch. ** The letters for the degeneracy codes are M (A, C), S (C, G), Y (C, T), H (A, C, and T), V (A, C, and G), B (C, G, and T), and N (A, C, G, and T).

**Table 2:** Amplification efficiency influenced by more than one nucleotide mismatch at 3’ end of the primer.

Nucleotide Mismatch	Amplification Efficiency	
GGGGTTGTAGGGTCGATAACGTGAGATC	Platinum, 100%	Takara, 100%
GGGGTTGTAGGGTCGATAACGTGAGA**AG**	0%	160%
GGGGTTGTAGGGTCGATAACGTGAGA**CG**	0%	87%
GGGGTTGTAGGGTCGATAACGTGAGA**GG**	0%	100%
GGGGTTGTAGGGTCGATAACGTGAGA**AA**	0%	100%
GGGGTTGTAGGGTCGATAACGTGAGA**CA**	0%	100%
GGGGTTGTAGGGTCGATAACGTGAGA**GA**	0%	100%
GGGGTTGTAGGGTCGATAACGTGAGA**AT**	0%	170%
GGGGTTGTAGGGTCGATAACGTGAGA**CT**	1%	138%
GGGGTTGTAGGGTCGATAACGTGAGA**GT**	0%	150%
GGGGTTGTAGGGTCGATAACGTGAG**TAG**	0%	100%
GGGGTTGTAGGGTCGATAACGTGAG**CAG**	0%	73%
GGGGTTGTAGGGTCGATAACGTGAG**GAG**	0%	100%
GGGGTTGTAGGGTCGATAACGTGAG**TCG**	0%	100%
GGGGTTGTAGGGTCGATAACGTGAG**CCG**	0%	72%
GGGGTTGTAGGGTCGATAACGTGAG**GCG**	0%	99%
GGGGTTGTAGGGTCGATAACGTGAG**TGG**	0%	85%
GGGGTTGTAGGGTCGATAACGTGAG**CGG**	0%	43%
GGGGTTGTAGGGTCGATAACGTGAG**GGG**	0%	81%
GGGGTTGTAGGGTCGATAACGTGAG**TAA**	0%	45%
GGGGTTGTAGGGTCGATAACGTGAG**CAA**	0%	34%
GGGGTTGTAGGGTCGATAACGTGAG**GAA**	0%	45%
GGGGTTGTAGGGTCGATAACGTGAG**TCA**	0%	40%
GGGGTTGTAGGGTCGATAACGTGAG**CCA**	0%	35%
GGGGTTGTAGGGTCGATAACGTGAG**GCA**	0%	29%
GGGGTTGTAGGGTCGATAACGTGAG**TGA**	0%	40%
GGGGTTGTAGGGTCGATAACGTGAG**CGA**	1%	18%
GGGGTTGTAGGGTCGATAACGTGAG**GGA**	2%	79%
GGGGTTGTAGGGTCGATAACGTGAG**TAT**	0%	52%
GGGGTTGTAGGGTCGATAACGTGAG**CAT**	0%	45%
GGGGTTGTAGGGTCGATAACGTGAG**GAT**	10%	100%
GGGGTTGTAGGGTCGATAACGTGAG**TCT**	9%	100%
GGGGTTGTAGGGTCGATAACGTGAG**CCT**	0%	0%
GGGGTTGTAGGGTCGATAACGTGAG**GCT**	0%	100%
GGGGTTGTAGGGTCGATAACGTGAG**TGT**	0%	55%
GGGGTTGTAGGGTCGATAACGTGAG**CGT**	0%	20%
GGGGTTGTAGGGTCGATAACGTGAG**GGT**	0%	100%
GGGGTTGTAGGGTCGATAACGTGA**TCGA**	0%	3%
GGGGTTGTAGGGTCGATAACGTGA**CGAA**	0%	14%
GGGGTTGTAGGGTCGATAACGTG**CTCGA**	0%	0%
GGGGTTGTAGGGTCGATAACGTG**GCGAA**	0%	4%

**Table 3:** Oligonucleotide primers for *Babesia* and *Theileria* used in this study.

	Primers	Nucleotide Sequences (5’–3’)	Ref.
PCR-1	*Babesia*-UP *	TAGTGACAAGAAATAACAATAC**A**GGGC**G**	This study
	*Theileria*-UP	TAGTGACAAGAAATAACAATAC**G**GGGC**T**	
	Ba/Th-DN	GCTTTCGCAGTAGTTCGTCTTTAACAA	
PCR-2	*Babesia*-UP	AATGTCTTGTAATTGGAATGATGG**T**	
	*Theileria*-UP	AATGTCTTGTAATTGGAATGATGG**G**	
	Ba/Th-DN	TTCGCAGTAGTTCGTCTTTAACAA	
PCR-3	Ba/Th-UP	CGCAAATTACCCAATCCTGACA	
	*Babesia*-DN	CAACTACGAGCTTTTTAACTGCAACAA**G**	
	*Theileria*-DN	CAACTACGAGCTTTTTAACTGCAACAA**T**	
PCR-4	*Babesia*-UP	AATTCCAGCTCCAATAGCGTATATTAAA**C**	
	*Theileria*-UP	AATTCCAGCTCCAATAGCGTATATTAAA**A**	
	Ba/Th-DN	GCTTTCGCAGTAGTTCGTCTTTAACAA	
PCR-5	*Babesia*-UP	TTCAAGCAGACTTTTGTCTTGAATA**C**	
	*Theileria*-UP	TCAAAGCAGGCTTTTGCCTTGAATA**G**	
	Ba/Th-DN	GCTTTCGCAGTAGTTCGTCTTTAACAA	
PCR-6	BJ1	GTCTTGTAATTGGAATGATGG	[21]
	BN2	TAGTTTATGGTTAGGACTACG	
PCR-7	PIRO-A	AATACCCAATCCTGACACAGGG	[20]
	PIRO-B	TTAAATACGAATGCCCCCAAC	

* UP indicates the upstream primer, and DN is for the downstream primer. Ba/Th: *Babesia*/*Theileria*. Text with bold indicates nucleotide mismatch.

## Data Availability

The data underlying this article are available in the article and in its online supplementary data.

## Supplementary Materials

The following supporting information can be downloaded at: https://www.mdpi.com/article/10.3390/genes15020215/s1, Supplementary Data are available at NAR Online: https://ngdc.cncb.ac.cn/databasecommons/database/id/6417. Table S1. The MIQE Guidelines: Minimum Information for Publication of Quantitative Real-Time PCR Experiments. Table S2. Amplification efficiency influenced by nucleotide mismatches at 5′end and the center of the primer. Table S3. Amplification efficiency influenced by nucleotide m ismatches at both of the upstream and downstream primers. Figure S1. Establishment of a highly sensitive FRET-qPCR system. Figure S2. Comparative evaluation of the amplification efficiency of Chlamydia pneumonia FRET-qPCR system using seven types of DNA polymerases Figure S3. Change of amplification efficiencies induced by nucleotide mismatches at both ends of the primers.
