# Supplementary material for: Exploring the Impact of Primer–Template Mismatches on PCR Performance of DNA Polymerases Varying in Proofreading Activity
Source: Genes (Basel). 2024 Feb 7;15(2):215. doi: 10.3390/genes15020215 (PMC10888005; doi:10.3390/genes15020215)
Supplement: Supplementary file 1 [file genes-15-00215-s001.zip › genes-2820991-supplementary.pdf]

Supplementary Table S1

MIQE checklist for authors, reviewers, and editors.[1]

Item to check	Importance		Item to check	Importance	
Experimental design			qPCR oligonucleotides		
Definition of experimental and control groups	E	Y	Primer sequences	E	Y
Number within each group	E	Y	RTPrimerDB identification number	D	N/A
Assay carried out by the core or investigator's laboratory?	D	Y	Probe sequences	D[4]	Y
Acknowledgment of authors' contributions	D	Y	Location and identity of any modifications	E	N/A
Sample			Manufacturer of oligonucleotides	D	Y
Description	E	Y	Purification method	D	N/A
Volume/mass of sample processed	D	Y	qPCR protocol		
Microdissection or macrodissection	E	N/A	Complete reaction conditions	E	Y
Processing procedure	E	Y	Reaction volume and amount of cDNA/DNA	E	Y
If frozen, how and how quickly?	E	N/A	Primer, (probe), Mg^{2+}, and dNTP concentrations	E	Y
If fixed, with what and how quickly?	E	N/A	Polymerase identity and concentration	E	Y

Sample storage conditions and duration (especially for FFPE[2] samples)	E	N/A	Buffer/kit identity and manufacturer	E	Y
Nucleic acid extraction			Exact chemical composition of the buffer	D	Y
Procedure and/or instrumentation	E	Y	Additives (SYBR Green I, DMSO, and so forth)	E	N/A
Name of kit and details of any modifications	E	Y	Manufacturer of plates/tubes and catalog number	D	Y
Source of additional reagents used	D	Y	Complete thermocycling parameters	E	Y
Details of DNase or RNase treatment	E	N/A	Reaction setup (manual/robotic)	D	Y
Contamination assessment (DNA or RNA)	E	Y	Manufacturer of qPCR instrument	E	Y
Nucleic acid quantification	E	Y	qPCR validation		
Instrument and method	E	Y	Evidence of optimization (from gradients)	D	N/A
Purity (A_{260}/A_{280})	D	N/A	Specificity (gel, sequence, melt, or digest)	E	Y
Yield	D	N/A	For SYBR Green I, C_q of the NTC	E	N/A
RNA integrity: method/instrument	E	N/A	Calibration curves with slope and *y* intercept	E	Y
RIN/RQI or C_q of 3′ and 5′ transcripts	E	N/A	PCR efficiency calculated from slope	E	Y
Electrophoresis traces	D	Y	CIs for PCR efficiency or SE	D	N/A

Inhibition testing (C_q dilutions, spike, or other)	E	NO	r^2 of calibration curve	E	Y
Reverse transcription			Linear dynamic range	E	Y
Complete reaction conditions	E	N/A	C_q variation at LOD	E	Y
Amount of RNA and reaction volume	E	N/A	CIs throughout range	D	NO
Priming oligonucleotide (if using GSP) and concentration	E	N/A	Evidence for LOD	E	Y
Reverse transcriptase and concentration	E	N/A	If multiplex, efficiency and LOD of each assay	E	N/A
Temperature and time	E	N/A	Data analysis		
Manufacturer of reagents and catalogue numbers	D	N/A	qPCR analysis program (source, version)	E	Y
C_qs with and without reverse transcription	D[3]	N/A	Method of C_q determination	E	Y
Storage conditions of cDNA	D	N/A	Outlier identification and disposition	E	N/A
qPCR target information			Results for NTCs	E	Y
Gene symbol	E	Y	Justification of number and choice of reference genes	E	N/A
Sequence accession number	E	N/A	Description of normalization method	E	N/A
Location of amplicon	D	Y	Number and concordance of biological replicates	D	Y

Amplicon length	E	Y	Number and stage (reverse transcription or qPCR) of technical replicates	E	Y
In silico specificity screen (BLAST, and so on)	E	Y	Repeatability (intraassay variation)	E	Y
Pseudogenes, retropseudogenes, or other homologs?	D	N/A	Reproducibility (interassay variation, CV)	D	N/A
Sequence alignment	D	N/A	Power analysis	D	N/A
Secondary structure analysis of amplicon	D	N/A	Statistical methods for results significance	E	N/A
Location of each primer by exon or intron (if applicable)	E	N/A	Software (source, version)	E	N/A
What splice variants are targeted?	E	N/A	C_q or raw data submission with RDML	D	N/A

1 All essential information (E) must be submitted with the manuscript. Desirable information (D) should be submitted if available. If primers are from RTPrimerDB, information on qPCR target, oligonucleotides, protocols, and validation is available from that source.

2 FFPE, formalin-fixed, paraffin-embedded; RIN, RNA integrity number; RQI, RNA quality indicator; GSP, gene-specific priming; dNTP, deoxynucleoside triphosphate.

3 Assessing the absence of DNA with a no–reverse transcription assay is essential when first extracting RNA. Once the sample has been validated as DNA free, inclusion of a no–reverse transcription control is desirable but no longer essential.

4 Disclosure of the probe sequence is highly desirable and strongly encouraged; however, because not all vendors of commercial predesigned assays provide this information, it cannot be an essential requirement. Use of such assays is discouraged.

Table S2. Amplification efficiency influenced by nucleotide mismatches at 5'end and the center of the primer

Nucleotide mismatch	Amplification efficiency	
**GGGGTTGTAGGGTCGATAACGTGAGATC**	**Platinum, 100%**	**Taraka, 100%**
GGGGTTGTAGGG**AGT**ATAACGTGAGATC	93%	100%
GGGGTTGTAGGG**GACT**TAACGTGAGATC	100%	100%
GGGGTTGTAGGG**CGTTA**AACGTGAGATC	87%	106%
**ACCTG**TGTAGGGTCGATAACGTGAGATC	100%	100%
**ACCTGA**GTAGGGTCGATAACGTGAGATC	93%	100%
**ACCTGACG**AGGGTCGATAACGTGAGATC	82%	113%
**ACCTGACGTCC**GTCGATAACGTGAGATC	0%	2%
**ACCTGACGTCCCGA**GATAACGTGAGATC	0%	0%
**ACCTGACGTCCCGACTA**AACGTGAGATC	0%	0%

*：Text with bold indicates nucleotide mismatches.

Table S3. Amplification efficiency influenced by nucleotide mismatches at both of the upstream and downstream primers

Nucleotide mismatch	Amplification efficiency	
**up : GGGGTTGTAGGGTCGATAACGTGAGATC*** **dn : GAGAGTGGTCTCCCCAGATTCAGACTA**	**Platinum, 100%**	**Takara, 100%**
up : GGGGTTGTAGGGTCGATAACGTGAG**TAG** dn : GAGAGTGGTCTCCCCAGATTCAGACT**T**	0%	19%
up : GGGGTTGTAGGGTCGATAACGTGAG**CAG** dn : GAGAGTGGTCTCCCCAGATTCAGA**AGT**	0%	20%
up : GGGGTTGTAGGGTCGATAACGTGAG**GAG** dn : **CTCTC**TGGTCTCCCCAGATTCAGACTA	0%	51%
up : GGGGTTGTAGGGTCGATAACGTGAG**GAG** dn : GAGAGTGGTCT**GAGAC**GATTCAGACTA	0%	80%
up : **ACCTG**TGTAGGGTCGATAACGTGAGATC dn : **CTCTC**TGGTCTCCCCAGATTCAGACTA	81%	78%
up : **ACCTG**TGTAGGGTCGATAACGTGAGATC dn : GAGAGTGGTCT**GAGAC**GATTCAGACTA	77%	80%
up : **ACCTG**TGTAGGGTCGATAACGTGAGATC dn : GAGAGTGGTCTCCCCAGATTCAGA**AGT**	0%	31%
up : **ACCTG**TGTAGGGTCGATAACGTGAGATC dn : GAGAGTGGTCTCCCCAGATTCAGACT**T**	74%	58%
up : GGGGTTGTAGGG**CGTTA**AACGTGAGATC dn : **CTCTC**TGGTCTCCCCAGATTCAGACTA	69%	44%
up : GGGGTTGTAGGG**CGTTA**AACGTGAGATC dn : GAGAGTGGTCT**GAGAC**GATTCAGACTA	49%	73%
up : GGGGTTGTAGGG**CGTTA**AACGTGAGATC dn : GAGAGTGGTCTCCCCAGATTCAGA**AGT**	0%	40%
up : GGGGTTGTAGGG**CGTTA**AACGTGAGATC dn : GAGAGTGGTCTCCCCAGATTCAGACT**T**	67%	50%
up : GGGGTTGTAGGGTCGATAACGTGA**TCGA** dn : GAGAGTGGTCTCCCCAGATTCAGA**AGT**	0%	1%
up : GGGGTTGTAGGGTCGATAACGTGAGATC dn : GAGAGTGGTCTCCCCAGATTCAGA**AGT**	0%	41%

* up indicates the upstream primer, and dn is for the downstream primer. Text with bold indicates a nucleotide mismatch.

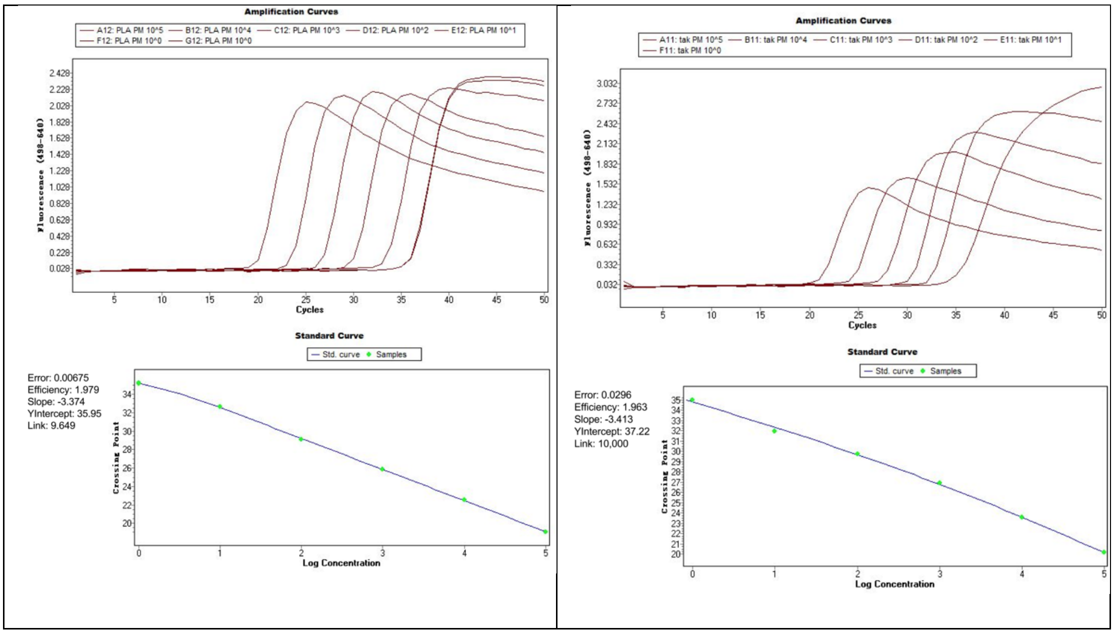

Figure S1. Establishment of a highly sensitive FRET-qPCR system. The established FRET-qPCR system can reliably amplify a single copy of 23S rRNA of *C. pneumoniae* per 20 µL reaction system while Platinum®*Taq*DNA polymerase (Invitrogen, USA, batch number: 10966-018, 8215130; left panel) and TaKaRa (Ex *Taq*®Hot Start Version, Takara, Japan, batch number: RR06WZ, RR006Q; right panel) were used. The amplification curves for both enzymes are shown the quantitative standards (10^5, 10^4, 10^3, 10^2, 10^1, 10^0 copies / reaction).

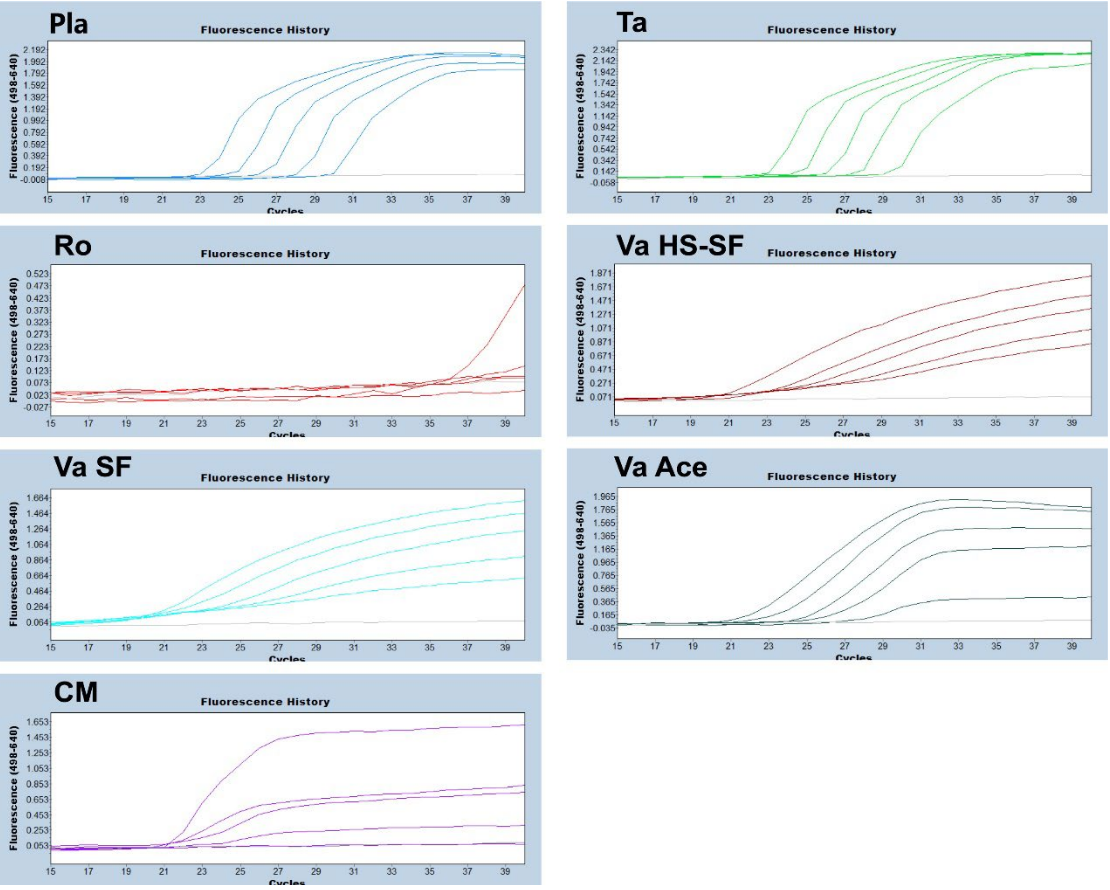

Figure S2. Comparative evaluation of the amplification efficiency of *Chlamydia pneumonia* FRET-qPCR system using seven types of DNA polymerases. Among seven DNA polymerase tested in *C. pneumoniae* FRET-qPCR, Platinum® *Taq*DNA polymerase (Invitrogen, USA, batch number: 10966-018, 8215130) and TaKaRa Ex *Taq*®Hot Start Version (Takara, Japan, batch number: RR06WZ, RR006Q) showed highly sensitive amplification of a single copy of the *C. pneumoniae* 23S rRNA. Efficient application was not observed while using five other DNA polymerases: FastStart *Taq* DNA Polymerase (Roche, USA, 12032902001, 13860081), Phanta™ HS Super-Fidelity DNA Polymerase (Vazyme, Germany, P502, 013041), Phanta™ Super-Fidelity DNA Polymerase (Vazyme, Germany, P501, 017031), Ace*Taq*™ HS DNA Polymerase (Vazyme, P401, 425031; Chimerigen Laboratories *Taq* DNA Polymerase), Chimerigen (Germany, TG102, D1208001). The quantitative standards of 10^0 to 10^4 of *C. pneumoniae* 23S rRNA were used to test PCR amplification system.

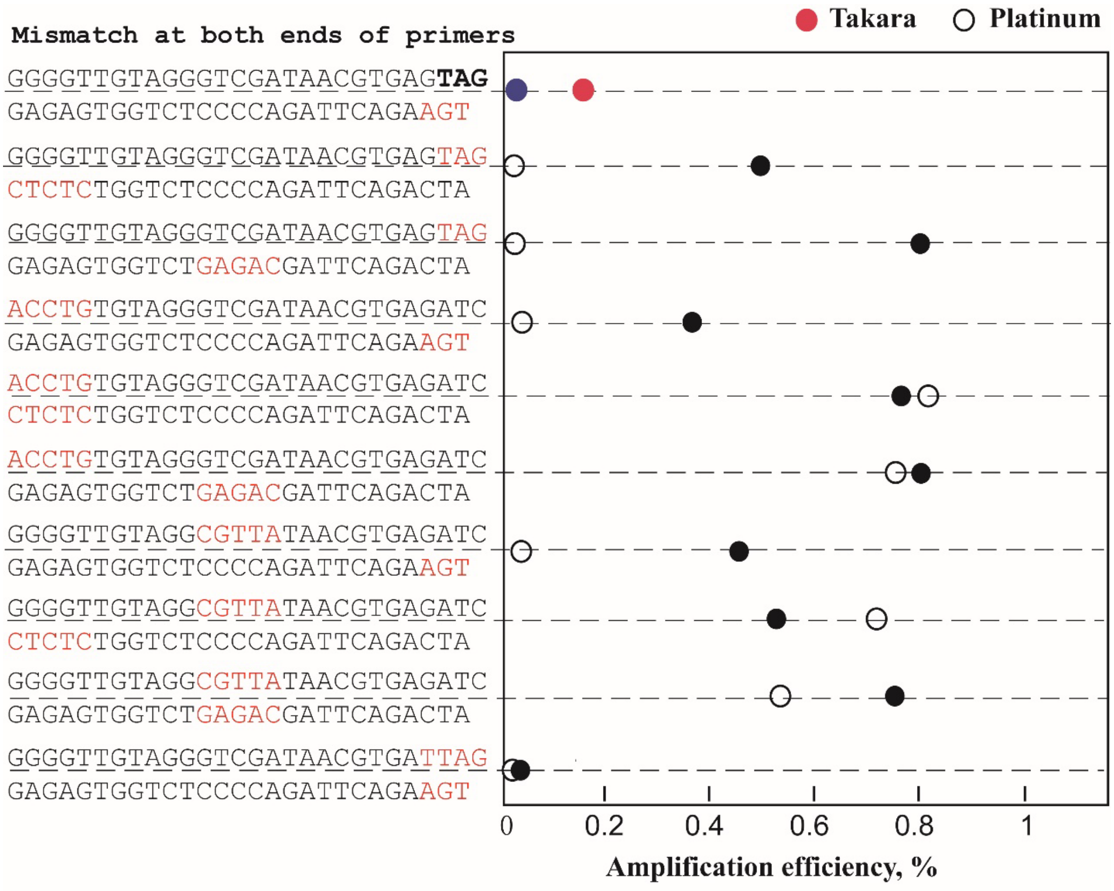

Figure S3. **Change of amplification efficiencies induced by nucleotide mismatches at both ends of the primers.** Ten copies of *Chlamydia* 23S rRNA were used to observe the change in the amplification efficiencies of PCRs induced by nucleotide mismatches at both ends of the primers. Text with a red font indicates a nucleotide mismatch between primers.
